# Draft Genome Sequence of the Growth-Promoting Endophyte *Paenibacillus* sp. P22, Isolated from *Populus*

**DOI:** 10.1128/genomeA.00276-14

**Published:** 2014-04-10

**Authors:** Anne M. Hanak, Matthias Nagler, Thomas Weinmaier, Xiaoliang Sun, Lena Fragner, Clarissa Schwab, Thomas Rattei, Kristina Ulrich, Dietrich Ewald, Marion Engel, Michael Schloter, Romana Bittner, Christa Schleper, Wolfram Weckwerth

**Affiliations:** a Department of Ecogenomics and Systems Biology, University of Vienna, Vienna, Austria; b Department of Microbiology and Ecosystem Science, University of Vienna, Vienna, Austria; c Johann Heinrich von Thünen Institute, Federal Research Institute for Rural Areas, Forestry and Fisheries, Institute of Forest Genetics, Waldsieversdorf, Germany; d Research Unit Environmental Genomics, Helmholtz Zentrum Munich, Neuherberg, Germany

## Abstract

*Paenibacillus* sp. P22 is a Gram-negative facultative anaerobic endospore-forming bacterium isolated from poplar hybrid 741 (♀[*Populus alba* × (*P. davidiana* + *P. simonii*) × *P. tomentosa*]). This bacterium shows strong similarities to *Paenibacillus humicus*, and important growth-promoting effects on *in vitro* grown explants of poplar hybrid 741 have been described.

## GENOME ANNOUNCEMENT

*Bacillus* is a phylogenetically heterogeneous taxon, and *Paenibacillus* was classified as a new genus in 1993 (1–3). The rod-shaped cells are motile, have peritrichous flagella, and show variable Gram staining. They form ellipsoidal endospores (4). Species of this genus are known to produce hormones that stimulate plant growth, like cytokinin (5), and antibiotic peptides as well as different (6) hydrolyzing enzymes, which are responsible for antagonistic behavior against many plant pathogens. Thus, many species of the genus have been described as plant growth-promoting bacteria. *Paenibacillus* sp. P22 was isolated by Ulrich et al. (7) from the poplar hybrid 741 [*Populus alba* × (*P. davidiana* + *P. simonii*) × *P. tomentosa*] (8). The phylogenetic analysis of the strain was based on 16S rRNA gene sequencing and showed that *Paenibacillus* sp. P22 has strong 16S rRNA gene sequence similarity to *Paenibacillus humicus* (99.5%) (7). Former experiments have shown that *in vitro* grown explants of hybrid 741 inoculated with *Paenibacillus* sp. P22 exhibited significantly more root growth and root length than noninoculated explants (7). The pure culture of the bacterial strain was grown under aerobic conditions on tryptic soy broth agar plates. The DNA extraction was performed with a DNA GeneJET gel extraction kit according to the manufacturer’s instructions. Application of the 454 GS FLX Titanium sequencing technology and sequencing of an 8-kb paired-end library resulted in 561,213 reads and 61,143,112 nucleotides. In an Ion Torrent PGM sequencing approach, 1,978,332 reads and 343,311,791 nucleotides were gathered. Consensus assembly using MIRA (9) yielded 5,443,257 bp in 297 contigs (>300 bp), with an overall GC content of 58%. Coding sequences (CDS) were predicted based on an in-house workflow that integrates *ab initio* predictions from Glimmer (10), Genemark (11), Prodigal (12), and Critica (13) with homology information derived from a BLAST search against NCBI nr (14). Noncoding RNAs were identified by tRNAscanSE (15), RNAmmer (16), and Infernal (17). Predicted CDS were compared to the databases InterPro (18), Swissprot (19), and trEMBL (19) for functional annotation and mapped to KEGG pathways.

The genome of *Paenibacillus* sp. P22 contains 5,224 protein-coding genes, 65 tRNAs, and 1 16S rRNA. Presence of tRNAs for all 20 proteinogenic amino acids as well as 31 out of 31 phylogenetic marker proteins (AMPHORA2 software) (20) that are essential in prokaryotes indicates an estimated completeness of the genome of about 99%. Further investigation of the metabolic capabilities of *Paenibacillus* sp. P22 yielded two particularly interesting findings. We found a gene encoding a nitrogenase (EC 1.19.6.1) for nitrogen fixation coinciding with the observation that *Paenibacillus* sp. P22 is able to grow without nitrogen in the medium (21). Accordingly, metabolite profiles of poplar plants which were inoculated with *Paenibacillus* sp. P22 showed a strongly altered C/N homeostasis as a result of the endophyte-plant interaction (21). Genes of the auxine-pathway were also detected, suggesting growth-promoting effects by hormone secretion. This finding was indeed confirmed by the detection of auxin in a metabolite profile of a *Paenibacillus* sp. P22 culture.

### Nucleotide sequence accession numbers.

The genome sequence of *Paenibacillus* sp. P22 has been deposited in the European Nucleotide Archive under the accession numbers CBRA020000001 through CBRA020000297.
